# Merging of Bi-Modality of Ultrafast Laser Processing: Heating of Si/Au Nanocomposite Solutions with Controlled Chemical Content

**DOI:** 10.3390/nano14040321

**Published:** 2024-02-06

**Authors:** Yury V. Ryabchikov, Inam Mirza, Miroslava Flimelová, Antonin Kana, Oleksandr Romanyuk

**Affiliations:** 1 HiLASE Centre, Institute of Physics of the Czech Academy of Sciences, Za Radnicí 828, 252 41 Dolní Břežany, Czech Republic; 2 Department of Analytical Chemistry, University of Chemistry and Technology, Prague Technická 5, 166 28 Prague, Czech Republic; 3 Department of Optical Materials, Institute of Physics of the Czech Academy of Sciences, Cukrovarnická 10, 162 00 Prague, Czech Republic

**Keywords:** ultrafast laser processing, silicon nanoparticles, plasmonic nanoparticles, Si-Au composite nanoparticles, nanocomposites, laser ablation, hyperthermia, photo-thermal therapy

## Abstract

Ultrafast laser processing possesses unique outlooks for the synthesis of novel nanoarchitectures and their further applications in the field of life science. It allows not only the formation of multi-element nanostructures with tuneable performance but also provides various non-invasive laser-stimulated modalities. In this work, we employed ultrafast laser processing for the manufacturing of silicon–gold nanocomposites (Si/Au NCs) with the Au mass fraction variable from 15% (0.5 min ablation time) to 79% (10 min) which increased their plasmonic efficiency by six times and narrowed the bandgap from 1.55 eV to 1.23 eV. These nanostructures demonstrated a considerable fs laser-stimulated hyperthermia with a Au-dependent heating efficiency (~10–20 °C). The prepared surfactant-free colloidal solutions showed good chemical stability with a decrease (i) of zeta (ξ) potential (from −46 mV to −30 mV) and (ii) of the hydrodynamic size of the nanoparticles (from 104 nm to 52 nm) due to the increase in the laser ablation time from 0.5 min to 10 min. The electrical conductivity of NCs revealed a minimum value (~1.53 µS/cm) at 2 min ablation time while their increasing concentration was saturated (~10^12^ NPs/mL) at 7 min ablation duration. The formed NCs demonstrated a polycrystalline Au nature regardless of the laser ablation time accompanied with the coexistence of oxidized Au and oxidized Si as well as gold silicide phases at a shorter laser ablation time (<1 min) and the formation of a pristine Au at a longer irradiation. Our findings demonstrate the merged employment of ultrafast laser processing for the design of multi-element NCs with tuneable properties reveal efficient composition-sensitive photo-thermal therapy modality.

## 1. Introduction

Ultrafast laser technologies reveal wide prospects for their employment in different scientific and application areas. They are used not only for material processing but also hide a large application potential, especially in the field of life sciences. One of the most promising applications is related to photo-thermal therapy (PTT) caused by laser-induced hyperthermia. For this purpose, a wide set of different classes of nanostructures such as plasmonic and magnetic metal nanoparticles (NPs), semiconductor-based NPs or natural and synthetic polymer NPs are demonstrated [1,2,3,4,5]. At the same time, similar laser sources are widely used for the fabrication of various nanomaterials in gaseous and liquid environments. Here, pulsed laser ablation in liquids (PLALs) is one of the simplest as well as versatile techniques to directly synthesize colloidal solutions of “green” nanostructures that can be employed for different applications such as biosensing, bioimaging, catalysis or theranostics [6,7,8,9,10]. Indeed, the easy preparation of colloids containing semiconductor, metallic or bimetallic NPs is successfully demonstrated using a large set of different laser sources [11,12,13,14,15,16,17].

However, the laser-based formation of semiconductor–metallic nanocomposites (NCs) with required performance is still a challenging task and is a very young direction of research in the laser–matter interaction field. Only recently, this easy way of merging semiconductor and metallic elements in one nanoparticle owing to the laser-assisted interaction was developed and demonstrated [18,19,20,21,22,23]. As a result, colloidal solutions containing surfactant-free compound nanostructures were formed and successfully tested for light-to-heat conversion [19] or surface-enhanced Raman scattering (SERS) biosensing. Such chemically pure nanomaterials containing several elements responsible for different specific actions can be very promising for multi-modal biomedical or catalytic applications. Nevertheless, their physico-chemical properties and mechanisms of the laser-assisted interaction between semiconductor and metallic species are not established yet. It significantly complicates a proper design and further laser-based fabrication of semiconductor–metallic NC with properties required for each specific task.

Ultrafast laser processing of materials significantly depends on used experimental conditions. Here, one of the key parameters of the PLALs is the laser energy fluence that considerably affects properties of laser-synthesized NPs [24,25,26,27]. In particular, higher laser energy leads to the growth of larger Au NPs while the size of Si NPs decreases at similar conditions [28] that were chosen here for the fabrication of NCs. Their choice was determined by the high prospects for these elements in the fields of nanothermometry [29,30,31] and life science [32,33,34,35]. However, contrary to the aforementioned cases, the laser fluence variation provokes neither changes of the size of Si/Au NCs nor modifications of their chemical content [19,20]. Apart from this, the amount of Si NPs in the liquid environment also considerably affects both the size distribution and the chemical composition of Si/Au NCs [20]. So, their larger concentration restricts the further growth of NCs increasing the Si content in them [20]. Evidently, the duration of the laser irradiation of a solid target immersed in a colloidal solution of NPs should also significantly affect the properties of NCs. Nevertheless, current state-of-the-art fails in properly addressing this research aspect of the ultrafast laser synthesis of nanomaterial colloidal solutions.

In this paper, we tend to achieve a synergy in a multi-modal employment of the ultrafast laser processing by merging the nanocomposite synthesis with their further hyperthermia application. The tuning of the laser ablation time resulted in the 6-fold enhancement of the efficiency of plasmonic properties leading to changes in the nanostructure colour and a reduction of the Si bandgap. Prolonged irradiation increased the NP concentration maintaining a low electrical conductivity of ~1.53 µS/cm. Our findings demonstrate that hydrodynamic size and ξ-potential can be tailored by adjusting ablation time and varying the Au content. We successfully employed the laser-generated NCs for ultrafast laser-induced hyperthermia whose efficiency was ~2–3 °C/min and was being affected by the Au content. The achieved findings successfully demonstrate the employment of multi-modal ultrafast laser processing in the fabrication of chemically pure semiconductor–metallic NCs with laser-controlled performance for their further applications.

## 2. Materials and Experimental Methods

**Figure 1:**
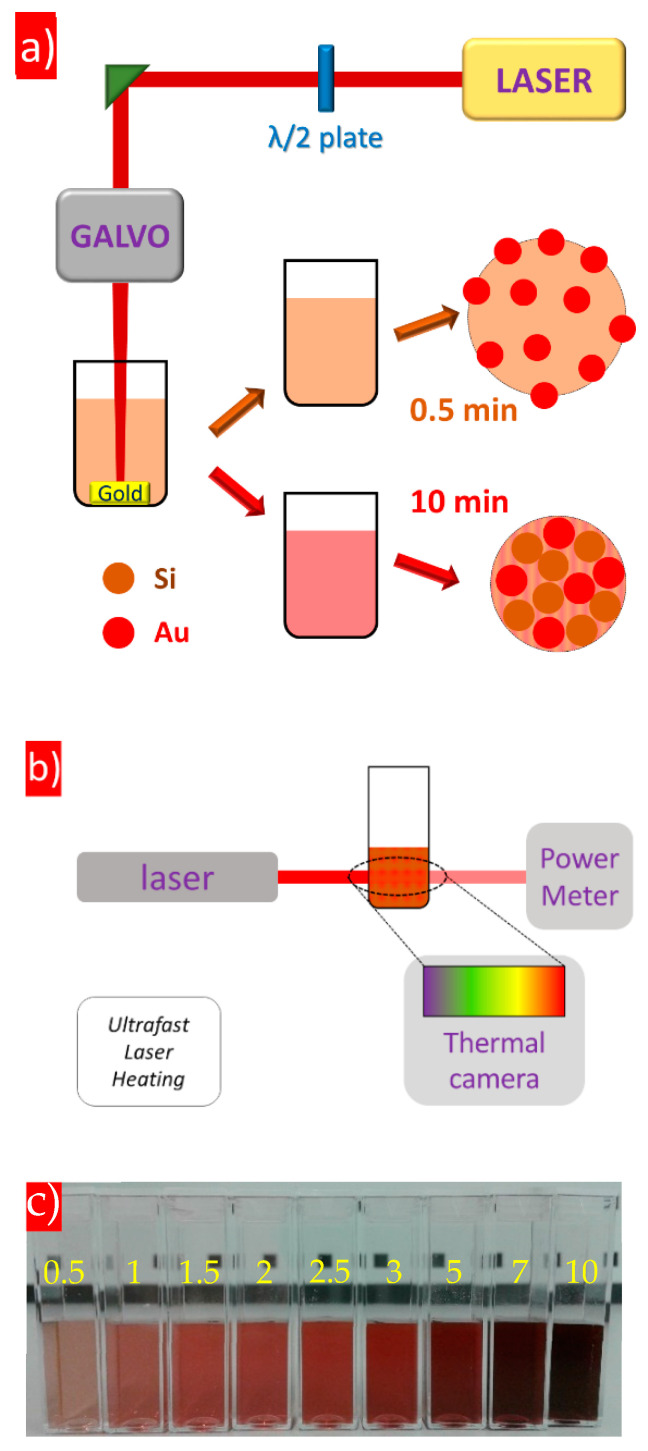
(**a**) A scheme of pulsed laser ablation experiments, (**b**) a scheme of laser-induced hyperthermia and (**c**) photo of colloidal solutions of Si/Au NCs formed at different laser ablation time (values indicate laser ablation time in minutes).

To prepare colloidal solutions of Si/Au NCs, a method of direct PLALs was employed by ablating a commercially available Au target (99.99% purity) immersed in laser-generated aqueous colloidal solutions (0.1 g/L concentration) of 30 nm Si NPs. The irradiation of an ultrafast laser (1030 nm, 6 ps pulse duration, 50 µJ/pulse, 10 kHz repetition rate, Gaussian shape) was focused on the target’s surface under a 3 mm water level into a 50 µm spot and scanned by a galvoscanner with 2 mm/s speed (Figure 1a). Laser ablation time was varied from 0.5 min to 10 min in order to change NC properties.

Optical properties of the laser-generated colloids of Si/Au NCs were studied by UV–Vis spectroscopy (Shimadzu 2700, Shimadzu Corporation, Kyoto, Japan) and Multi-Angle Dynamic Light Scattering (MA-DLS) (Malvern Ultra Size). The latter method allowed us to obtain a hydrodynamic size and concentration of NCs as well as ξ-potential and conductivity of the corresponding colloidal solutions. Prior to the measurements, the solutions were 10-fold diluted in order to prevent concentration-related signal distortions.

The chemical composition of Si/Au nanostructures was studied using inductively coupled plasma mass spectrometry (ICP-MS). For this purpose, aliquots (500 µL) of the samples were digested with 2.5 mL aqua regia (≥65% nitric acid, Analpure^®^ grade, Analytika spol. s.r.o., Prague, Czech Republic; 30% hydrochloric acid, Suprapur^®^ grade, Merck, Darmstadt, Germany) in a microwave-assisted digestion system (Speedwave 4, Berghof, Eningen unter Achalm, Germany) for 10 min at 190 °C. The digests were diluted with ultrapure water (18.2 MΩ·cm, Millipore, Bedford, MA, USA) to 50 mL after addition of 20 µg/L Rh as internal standard (1000 ± 2 mg/L Rh, Analytika spol. s.r.o., Czech Republic). The digests were subjected to an ICP-MS (Elan DRC-e, Perkin-Elmer, Concord, ON, Canada). Quantification was carried out via external calibration. Standard solution of Au (1000 ± 2 mg/L; Analytika spol. s. r.o., Czech Republic) was used to prepare calibration solutions in the range of 0–5 mg/L.

Structural properties of the NCs were evaluated by the following X-ray techniques. Firstly, grazing incidence X-ray diffraction (GIXRD) for Si/Au NCs coated on amorphous SiO_2_ substrate was performed using Rigaku Smartlab X-ray diffractometer at a critical angle of ~0.3 degree. Secondly, atomic composition of NCs surfaces (on Cu substrates) was investigated with X-ray photoelectron spectroscopy (XPS) technique. XPS spectra were detected by an AXIS-Supra photoelectron spectrometer (Kratos Analytical Ltd., Manchester, UK) using monochromatized Al K_α_ radiation (1486.6 eV, 300 W, analysed area of 0.7 × 0.3 mm^2^). The samples were prepared by depositing an aqueous solution of Si-based NPs onto a Cu substrate via drop casting. Subsequently, the samples were air-dried before being introduced into the XPS chamber for analysis. The X-ray incidence angle and photoelectron emission angle were 36° and 90° in respect to the surface plane, respectively. The high-resolution core level spectra were recorded with a pass energy of 10 eV, with an overall energy resolution of 0.45 eV and an energy step of 0.1 eV. During the measurements, a low energy electron flood gun was used for neutralization of charge on a surface. Measured spectra were calibrated to the position of Au 4f^7/2^ at 84.0 eV and C 1s at 285.0 eV. Atomic concentrations were computed by analysing the integrated core level peak areas. The Shirley’s electron inelastic background was subtracted from the spectra. We employed the ESCApe software (Kratos Analytical Ltd., Manchester, UK), utilizing the incorporated atomic sensitivity factors. Spectra were fitted by using the KolXPD 1.7.0.8 software by Voigt function doublets. The energy differences between the spin–orbit split components were fixed to 0.6 eV (Si 2p^1/2^–Si 2p^3/2^) and to 3.67 eV (Au 4f^5/2^–Au4f^7/2^). The spin–orbit split component area ratios were also kept fixed to 1.9 (for Si 2p) and 1.3 (for Au 4f).

Ultrafast laser heating of Si-based NPs was carried out using a laser source with the ultrashort pulse duration (35 fs) operated at 800 nm with a 1000 Hz repetition rate and 1.37 W power. The unfocused laser beam (~10 mm diameter) irradiated 1 mL of colloidal solutions of Si/Au NCs placed in a plastic cuvette (Figure 1b). The power of the laser beam, which was transmitted through a cuvette, was measured by a power meter. The temperature evaluation of the colloidal solutions was detected by a thermal camera Bosch GTC400C which had 50 mK thermal sensitivity.

## 3. Results and Discussion

Figure 1c shows colloidal solutions of Si/Au NCs prepared at different laser ablation times (0.5–10 min) indicating a considerable change of the colour of the solutions. Pure Si NPs of a brownish colour obtained a pink shade due to the interaction with emitted Au nanoclusters becoming deep red for NCs prepared at a longer ablation time. This visible transformation can occur due to changes of different properties of NPs in colloidal solutions such as their size, concentration, chemical composition or optical band gap.

**Figure 2:**
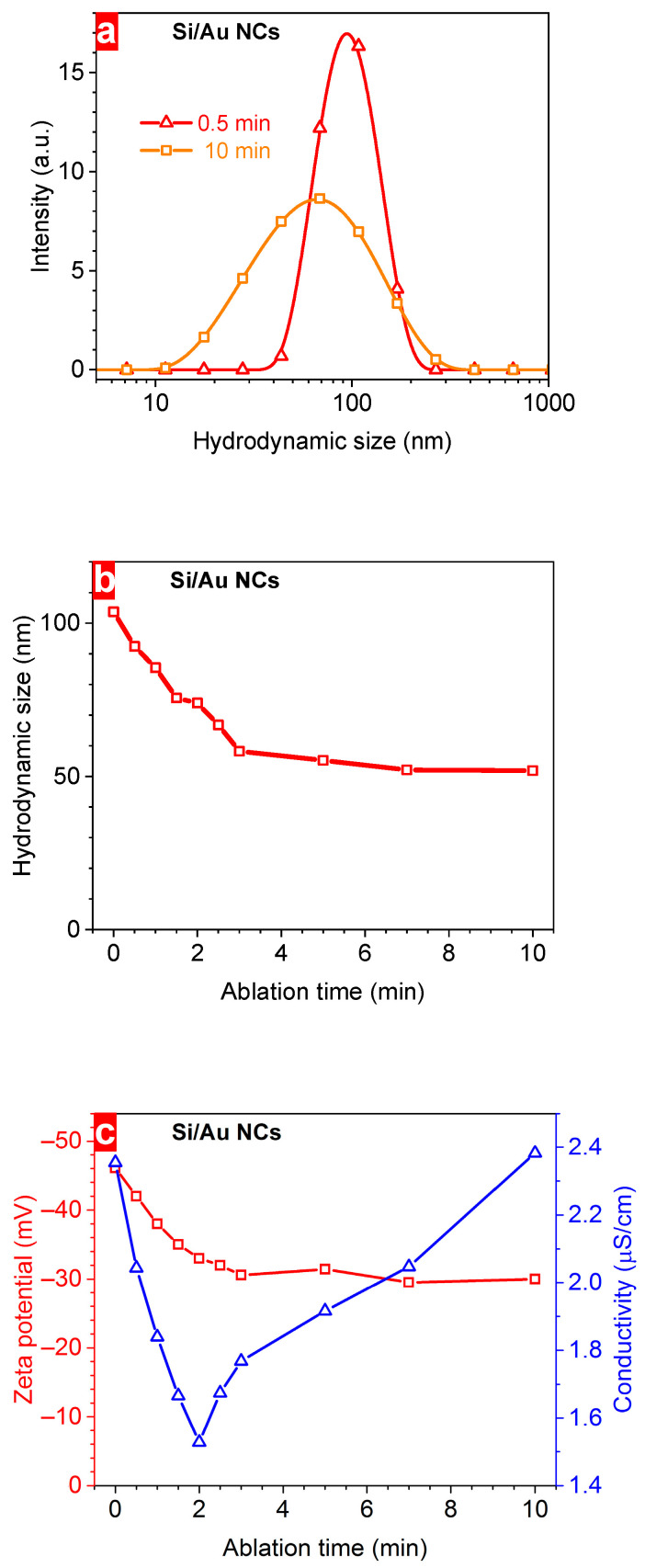
(**a**) MA-DLS size distributions of Si/Au NCs prepared at 0.5 min and 10 min laser ablation time, (**b**) a dependence of their hydrodynamic size on the laser ablation time, and (**c**) ξ-potential and conductivity values of their colloidal solutions.

To determine the hydrodynamic size of Si-based NPs, the corresponding colloidal solutions were studied by means of MA-DLS. One can see that the size decreased from 104 nm to 52 nm with the increase in the ablation time from 0 min (Si NPs) to 10 min (Si/Au NCs) (Figure 2a,b). It is known that the hydrodynamic size of NPs, reflecting the diffusion ability of nanomaterials, is larger than their physical dimensions due to the attachment of solvated water molecules. The following reasons can be responsible for the observed changes of the hydrodynamic size: (i) the decrease in the physical dimension of the NPs during the laser irradiation due to the laser-induced fragmentation or (ii) the decrease in the solvated shell due to some variations in chemical properties of NCs caused by the change of their content. In the last case, the chemical content of the NCs changes with the laser ablation time due to the interaction of Si NPs with the ablated gold nanoclusters and dissolved oxygen molecules. Longer laser irradiation can provoke the increase in the number of Si-O, Au-O and Si-Au bonds as shown below by XPS. These bonds might provoke the charge redistribution in NCs leading to reductions in the charged solvated shell of NPs.

Besides the hydrodynamic size, the ξ-potential value of the colloidal solutions is also decreased with the increase in the laser ablation time (Figure 2c). Indeed, the colloidal solution of pure Si NPs possessed −46 mV of ξ-potential value while Si/Au NCs formed at 10 min ablation time had only −30 mV. Thus, the increase in the ablation time provoked some deterioration of the chemical stability of colloidal solutions due to more favourable aggregation of Au nanostructures. It is also worth noticing that the Si/Au NCs possessed good long-term stability when being kept at normal ambient conditions. The properties of the colloidal solutions just changed slightly for NCs prepared at 5–7 min. Longer irradiation also induced modifications of the electrical conductivity of Si/Au NCs colloidal solutions (Figure 2c). Firstly, it was decreased from ~2.4 µS/cm (pure Si NPs) to ~1.5 µS/cm (Si/Au NCs prepared at 2 min) followed by a further increase to ~2.4 µS/cm for plasmonic nanostructures formed at 10 min. Firstly, at a lower irradiation time, it is assumed that nanostructured Au is either grafted to the surface of Si NPs or incorporated inside them during the laser-assisted fragmentation and further regrowth of the NPs. These metallic nanoclusters can serve as a trap of charge carriers reducing the electrical conductivity of the NCs. Moreover, the mobility of charge carriers in smaller Si nanostructures can be lower due to the quantum confinement effect. Secondly, starting from 2 min of ablation time, the amount of nanostructured Au can exceed a critical value forming large Au nanodomains that decreases the resistivity of formed NCs. Thus, such behaviour of the electrical conductivity can indicate a change in the structure of NPs at this point. The growing conductivity of Si/Au NCs can be used to increase the efficiency of biochemical or electrochemical sensors due to an electron transfer from a specific bio-layer to the surface of electrodes [36,37].

**Figure 3:**
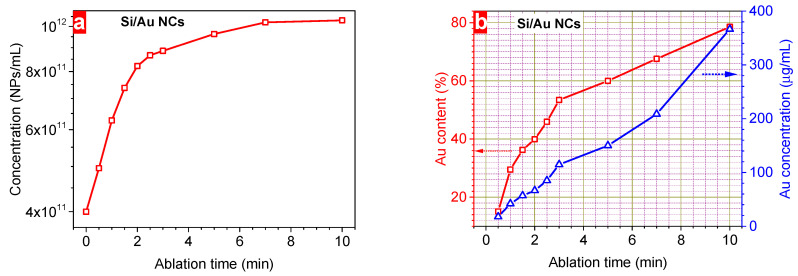
(**a**) dependence of the concentration of Si/Au NCs on the laser ablation time, (**b**) relative gold content (on the left) and absolute gold concentration (on the right) in Si/Au NCs prepared at different laser ablation times according to ICP-MS results.

To estimate the amount of NPs we performed the measurements using the DLS technique that allowed us to determine the number of particles in a volume unit. One can see that firstly the concentration increased with the ablation time from ~4 × 10^11^ NPs/mL for pure Si NPs to ~10^12^ NPs/mL at 7 min (Figure 3a). Starting from 4 to 5 min, the number of NPs changed slightly with the irradiation time. This saturation can be due to the considerable optical losses of the laser irradiation before reaching the target surface; it can be associated with the modification of the NP composition followed by the change of their absorbance. Indeed, a larger ablation time increased the Au concentration from ~20 µg/mL at 0.5 min to ~385 µg/mL according to ICP-MS measurements indicating considerable changes of the Si/Au ratio (Figure 3b). Indeed, the concentration of Au mass in solutions varied from ~15% (0.5 min) to ~79% (10 min). Thus, we determined for the first time, the exact chemical composition of Si/Au NPs prepared by PLALs depending on the irradiation time. These data will help to choose an appropriate ablation duration in order to synthesize NCs with required Au content reflecting plasmonic properties.

**Figure 4:**
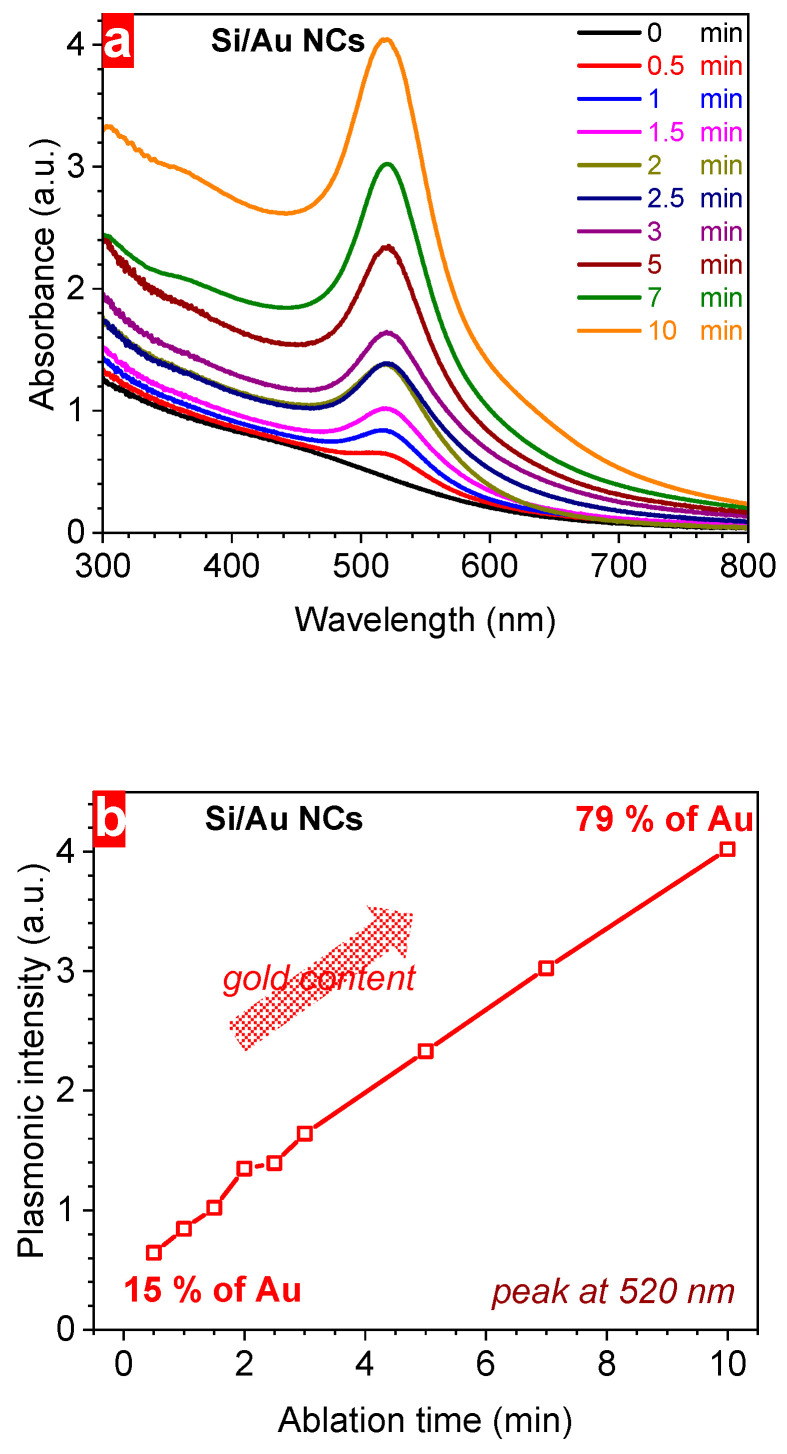

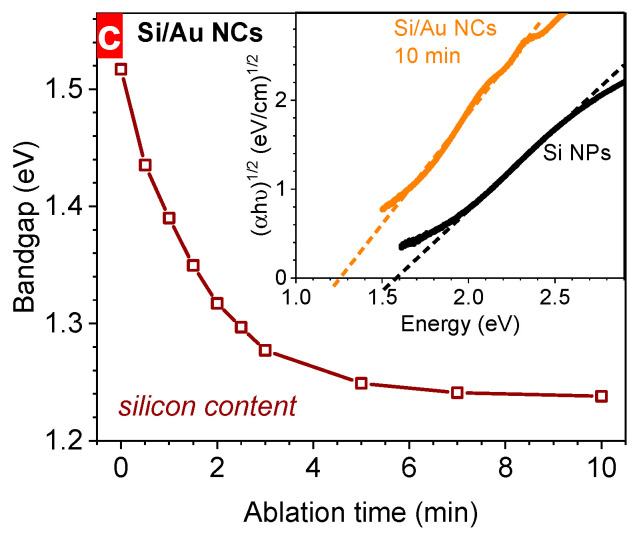
(**a**) absorbance spectra of Si/Au NCs at different ablation times, (**b**) a dependence of the plasmonic maximum efficiency on the laser ablation time, and (**c**) laser ablation time-dependent bandgap of Si/Au NCs (inset represents Tauc plot for Si NPs and Si/Au NCs prepared at 10 min ablation time).

In order to reveal plasmonic properties in Si/Au NPs, their absorption spectra were investigated using UV–Vis spectroscopy depending on the laser ablation time (Figure 4a). It is worth noticing that pure Si NPs have no specific features related to plasmonic properties. However, ablation of the Au target immersed in colloidal solution of Si NPs considerably changes their optical properties. Indeed, this study shows the appearance of plasmonic properties in Si NPs whose intensity increases with the irradiation duration (Figure 4a,b). Ablation of the Au leads to the emission of nanoclusters that can interact with Si NPs forming Si/Au NCs. Longer ablation increases the amount of ablated noble metal nanoclusters, thus, increasing Au content in NCs. The concentration of metallic nanostructures influences the intensity of the plasmonic peaks of Si/Au NCs. Hence, one can easily control the efficiency of the plasmonic properties in semiconductor nanostructures by choosing an appropriate irradiation time (Figure 4b). The laser-controlled plasmonic properties of Si/Au NCs can be employed for sensing applications using the surface enhancement of optical signals. Indeed, it has already been shown that such NCs are effective nanosensors for bacteria detection using SERS with the efficiency controlled by the Au/Si ratio. Considering the time-dependent chemical composition of NPs (Figure 3b), one can easily design and manufacture Si/Au NCs with required metal content and corresponding plasmonic efficiency.

In addition, the absorbance spectra can also give us the information on the bandgap value of the nanostructures. For this purpose, the experimental spectra were fitted by a sum of Gaussian and polynomic curves in order to obtain a perfect coincidence with the experimental data. Afterwards, the curve related to the semiconductor structure polynomic part was replotted using a Tauc plot [38]:

αhυ~(hυ − E_g_)^n^ (1)

here **α**—absorption coefficient, **υ**—light frequency, **E_g_** optical bandgap value. Parameter **n** differs for materials depending on the type of electronic transitions: ½ for direct allowed transitions, 3/2 for direct forbidden transitions, 2 for indirect allowed transitions and 3 for indirect forbidden transitions [39]. In the case of Si-based NPs, this parameter is defined as 2 due to the allowed indirect electron transitions between the valance and conduction bands. Thus, the intersection of the experimental curves with the x-axis plotted in (αhυ)^1/2^ versus hυ coordinates will give corresponding bandgap values. The inset of Figure 4c depicts these plots for initial Si NPs and Si/Au NCs formed at 10 min ablation time. It gives the following values of the Si-based NPs bandgap that decreases from 1.55 eV for pure Si NPs to 1.23 eV for Si/Au NCs with 79% of Au reflecting the changes of either their size or bandgap structure.

Changes in the bandgap of semiconductor materials can occur due to different reasons. Firstly, the size variation of the nanostructures affects their bandgap value due to the quantum confinement effect. But in this case, narrowing of the Si bandgap corresponds to increasing the size of the nanostructures according to the following formula [40,41]:

(2) $$E_{g}\left(d\right)=1.12+\frac{3.73}{d^{1.39}}$$

here **d** is the size of the Si nanostructures and **E_g_** is their optical bandgap values. However, this suggestion contradicts to the observed size decrease in Si/Au NCs (Figure 2b) [20,42]. Another reason for the bandgap modification can be associated with the formation of the shallow/deep donor/acceptor electronic states in the semiconductor bandgap playing a role of charge carrier traps. It is known that Au can introduce both acceptor (E_c_ − 0.55 eV) and donor (E_v_ + 0.35 eV) levels in the bandgap of bulk Si [43,44]. In our case, the bandgap narrowing for Si/Au NCs prepared at 10 min was ~0.32 eV which is close to the aforementioned donor electronic states. Here, one can hypothesize that laser-assisted synthesis of Si/Au nanostructures can form donor electronic states in the Si NPs bandgap. Moreover, donor electronic states can have either neutral or positive charge states [43] that can also lead to lowering of the ξ-potential (Figure 2c). Thus, the achieved findings using the UV–Vis spectroscopy give us information on the tuning of plasmonic efficiency and possible changes in the semiconductor structure due to the laser-assisted bandgap modification.

**Figure 5:**
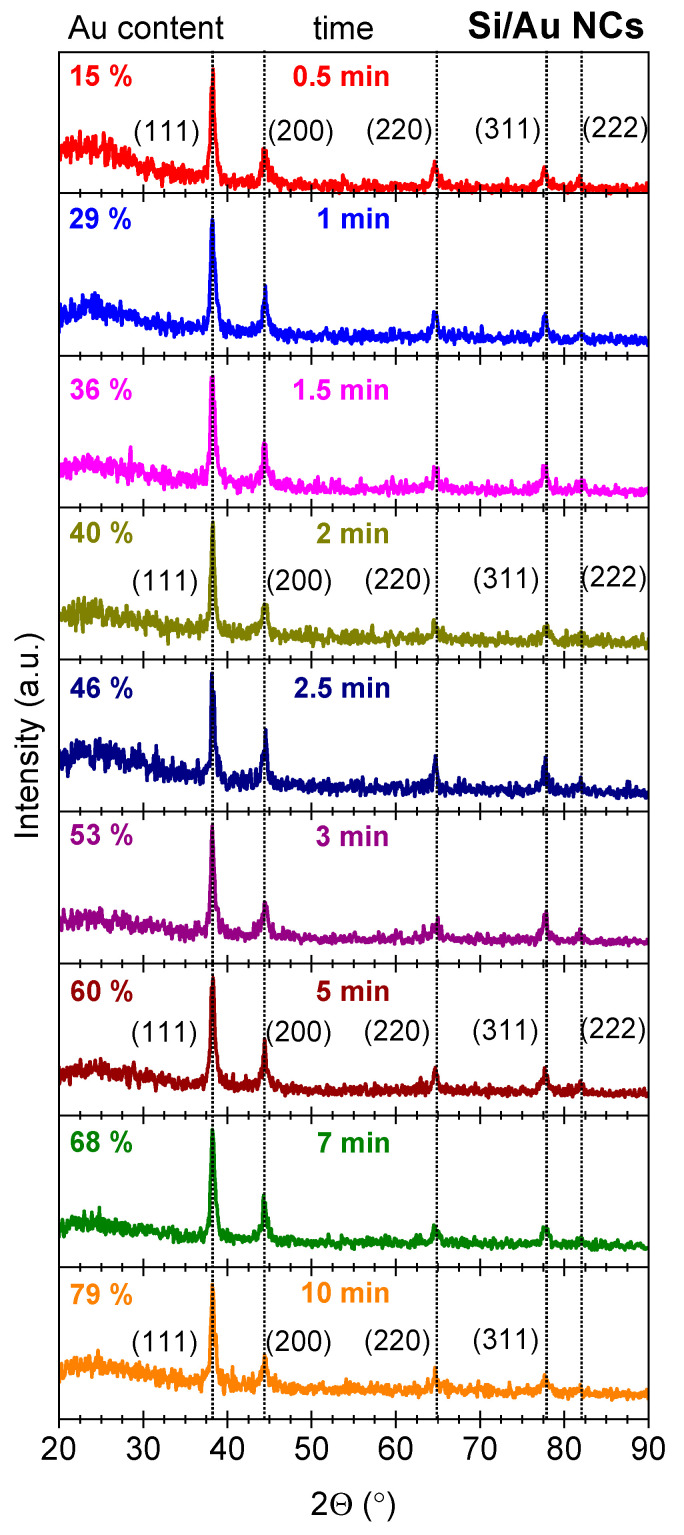
GIXRD patterns of Si/Au NCs formed at different laser ablation time.

To clarify the structure of laser-generated nanostructures, Si/Au NCs formed at different laser ablation times were studied by GIXRD. It is evident that these nanostructures possessed a multi-crystalline Au nature regardless of the time of laser irradiation, showing the reflexes at 38.3°, 44.5°, 64.7°, 77.8° and 81.9° (Figure 5). According to an XPS database (RRUFFID = R070279), the following crystalline planes corresponding to Au were identified (111), (200), (220), (311) and (222) (the corresponded interplanar distances are 2.350 Å, 2.035 Å, 1.439 Å, 1.227 Å and 1.175 Å), and they are in good agreement with our previous research. One can see the absence of sharp reflections related to the crystalline Si for the NCs synthesized at any laser ablation time. Thus, it points to the amorphous nature of Si in this type of nanostructures. However, the enhanced signal intensity around 20°–25° indicates the presence of some amount of SiO_2_ [45]. It is worth noticing that initial Si NPs revealed both amorphous (a wide peak at 2Θ = 23°) and polycrystalline phases with the following crystalline phases: (111), (220) and (311) [46,47].

**Figure 6:**
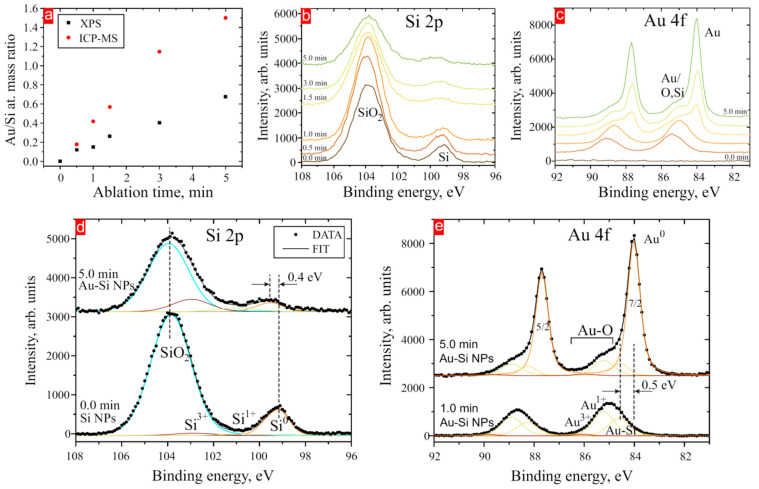
(**a**) The measured mass concentration ratio of Au/Si for varying ablation times. XPS spectra of (**b**) Si 2p and (**c**) Au 4f acquired at different ablation durations and high-resolution XPS spectra of (**d**) Si 2p and (**e**) Au 4f.

In order to reveal a chemical and electronic state of the NCs as well as their elemental composition, NPs were studied by XPS. Figure 6a shows the Au/Si mass ratio derived from the corresponding core level peak areas (XPS) and from the ICP-MS measurements. Qualitatively, both dependencies possess a similar trend showing an increase in the ratio with the ablation time. However, the ratio was larger for ICP-MS than for XPS measurements. This can be explained by the distinct surface sensitivities of both methods. XPS, with its higher surface sensitivity, and the ICP-MS method, which analyses volume content, are expected to yield different surface Si/Au and surface with bulk Si/Au ratios. This discrepancy is particularly notable for NP sizes larger than the information depth of XPS. In Figure 6a, more Au was measured by ICP for larger NPs, whereas some Au content was not detected by XPS due to the limitations of the information depth.

Figure 6b,c show the ablation–time-dependent XPS spectra adjusted along the intensity axis for clarity. The Au 4f consists of a 7/2 and 5/2 spin–orbit split peak doublet while the 3/2 and 1/2 components are not well resolved in Si 2p spectra. One might propose that such broadening is linked to the defective structure of Si-Si NPs, as evidenced by the measured FWHM of Si 2p^3/2^ for monocrystalline Si (001), which was 0.4 eV, compared to the broader FWHM of about 1.0–1.2 eV in Si^0^. Broader FWHMs typically correspond to an amorphous or defective structure of Si. These findings align with the measured band gap of ~1.5 eV in the 0 min Si NP sample, which is close to the band gap of 1.7 eV for amorphous Si. However, due to the relatively weak Si_0_ component intensity, no discernible dependence of FWHM of the Si^0^ component on ablation time was observed. The spectra at 0 min correspond to the reference Si-Si NPs sample excluding the presence of Au-Au NPs (the Au 4f peak was absent). For Si 2p, two distinct peaks were resolved in the XPS spectra corresponding to Si-Si bonds and Si oxide bonds. In the case of Au 4f peaks, Au-Si(O) peaks were resolved for short ablation periods (<1 min), while peaks associated with Au-Au bonds were observed for a longer ablation time.

The core-level peaks were deconvoluted into components, with representative fits depicted in Figure 6d,e. The fitted binding energy positions are combined and presented in Table 1. In the case of Si-Si NPs (0 min spectra), an elemental Si^0^ component and various oxidation states of Si oxides (Si^1+^, Si^3+^, and Si^4+^/SiO_2_) were resolved, with a corresponding chemical shift of 1.5 ± 0.1 eV, 3.5 ± 0.1 eV, and 4.5 ± 0.1 eV with respect to the Si^0^ position. It was also found that the area associated with Si sub-oxide peaks increased with the ablation time. The observed 0.4 eV shift between Si^0^ components might indicate the formation of Au-Si bonds, whereas the position of Si-O components remains constant. In the Au 4f spectra (Figure 6c), the Au-Si components were also identified, featuring chemical shifts of 0.5 eV. This shift is comparable to the Si^0^ shift for silicide and is in good agreement with the literature value of 0.7–0.8 eV for Au-Si [48].

Previous XPS studies on Au/Si interfaces revealed positive binding energy shifts in both Au 4f and Si 2p peaks indicating the formation of Au-Si bonds [49]. Specifically, the positive shifts in Si 2p peaks were attributed to the lower electronegativity of Si leading to the electron density transfer towards Au. Meanwhile, the positive shift in the Au 4f peaks was explained by the depletion of Au d-electrons. Distinct silicide stoichiometries encompassing Si-rich and Au-rich phases were discerned on pristine Si substrates through XPS [46]. Subsequent annealing led to shifts and splitting of the Au 4f and Si 2p peaks as a result of the phase separation into Au-rich and Si-rich silicide phases. Chemical shifts of approximately 0.7–0.8 eV for Au 4f and 0.6 eV for Si 2p were quantified for Au-Si bonds permitting the differentiation between metallic Au^0^ and its oxidized states, such as Au^1+^ and Au^3+^ [50]. Notably, the positive chemical shifts observed in oxide species exceeded those observed in Au silicide [51,52]. Furthermore, two oxidation states of Au^1+^ with a positive chemical shift of 1.2 eV and Au^3+^ with a shift of 2.0 eV were resolved. These findings suggest that Si/Au NPs underwent initial oxidation during brief ablation times. The Au^0^ component of Au NPs at 84 eV was elevated for longer ablation durations. In this context, both Si/Au NPs and Au-Au NPs could be formed simultaneously or, alternatively, complex Si-Au shell/Au-Au core hybrid NPs may have formed. However, at present, one cannot distinguish between these two possible structures. It is worth noticing that the concentration of Au-Au NPs was increased with prolonged ablation time. However, neither the Si-O nor Si-Au fraction of the Si phases was resolved by GIXRD.

**Figure 7:**
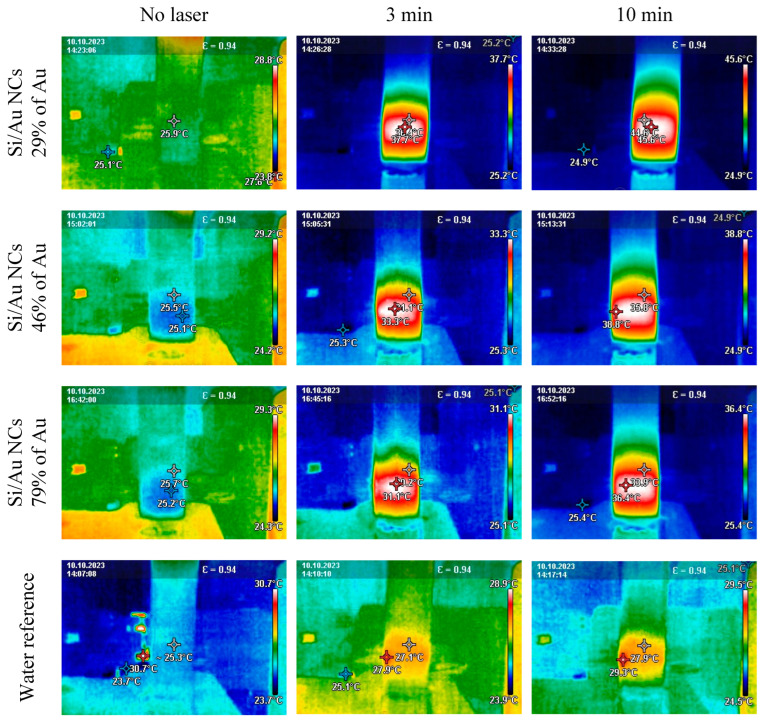
Ultrafast laser heating images of Si/Au NCs with different gold content at different irradiation durations.

To assess the prospect of Si/Au NCs for applications where laser-induced heating plays an important role (e.g., PTT), the colloidal solutions (1 mL) were irradiated by an fs laser for 10 min at 1.34 W power in the middle of the solution volume. In all cases, a drop of the laser power was detected after placing the samples under the laser beam. Here, the most pronounced power changes (~50%) were observed for all Si/Au NPs while pure water showed only ~15% of the power decrease. Thus, nanostructures contributed to considerable power losses due to the absorption of the laser irradiation that can be further converted into the heat. Indeed, monitoring of the laser-irradiated samples showed significant changes of their temperature depending on their chemical composition and irradiation duration (Figure 7). Firstly, all laser-unaffected solutions possessed a homogeneously distributed temperature (25 °C) in the whole volume (Figure 7, no laser). Then, the temperature started to increase after switching on one of the laser pulses. One can also mention a non-homogeneous heat distribution in the solutions showing the hottest area at the laser beam location surrounded with ~20% colder region (Si/Au NCs with 15% of Au irradiated at 10 min). It is worth noticing that the laser irradiation provoked just a slight temperature change of the pure distilled water while significant heating was observed for semiconductor–metallic NCs depending on their chemical content (Figure 7).

**Figure 8:**
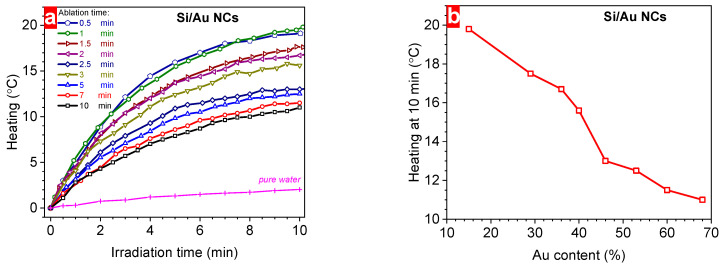
(**a**) Laser irradiation time-dependent heating of Si/Au NCs prepared at different ablation times, (**b**) the dependence of the maximum heating (at 10 min laser irradiation) on the gold content of Si/Au NCs.

Figure 8a depicts the heating dynamic of aqueous solutions of Si/Au NCs formed at a different laser ablation time depending on the duration of the laser irradiation that provoked light-to-heat conversion. As it was mentioned above, pure distilled water was heated insignificantly (~2 °C) by irradiating it for 10 min using an fs laser. However, the presence of Si/Au NCs in the liquid medium considerably changed the speed of the laser-induced heating of the solutions (Figure 8a). Indeed, Si/Au NCs formed at 1 min ablation time (with 29% of Au content) showed a one order of magnitude higher heating efficiency (~20 °C) as compared to pure water. The increase in the laser ablation duration provoked the reduction of the maximum temperature of the colloidal solutions reaching ~10 °C for nanostructures formed at a 10 min ablation time (Figure 8a). Considering the ablation-dependent variation of the chemical composition (Figure 4b), one can observe a monotonic decrease in the laser-induced temperature of the colloidal solutions with the increase in the Au content in NCs (Figure 8b). The change of the stimulated hyperthermia efficiency can be related to the variation of the thermal capacity of the NCs due to the modification of their structure and chemical composition. Indeed, Au has a higher value of the molar heat capacity than Si (25.42 J·mol^–1^·K^–1^ and 19.79 J·mol^–1^·K^–1^ at 25 °C, respectively). Hence, the increase in the Au content can lead to a higher thermal capacity value of Si/Au NCs, decreasing the maximum temperature value. Table 2 summarizes the found parameters of Si/Au NCs depending on the used laser ablation time. Thus, the achieved findings demonstrate high prospects for Si/Au NCs for the PTT applications due to content-controlled hyperthermia efficiency coupled with their plasmonic sensing modality.

## 4. Conclusions

In summary, ultrafast laser processing is employed for both the synthesis of colloidal solutions of semiconductor–metallic NCs and for their laser-induced hyperthermia. Longer laser ablation time decreases the hydrodynamic size (from 104 nm to 52 nm) and ξ-potential (from −46 mV to −30 mV) accompanied with the increase in their concentration till ~10^12^ NPs/mL. It also provokes stronger plasmonic properties of NCs due to the noble metal content being increased from 15% to 79%. The decrease in the bandgap values from 1.55 eV to 1.23 eV can be associated with the formation of deep donor electronic states in the semiconductor bandgap. All NCs reveal a polycrystalline Au structure with different phases of Au and Si depending on the ablation time. In particular, Au-Au, Au-O, Si-Si, Si-Au, and Si-O bonds were resolved by XPS. The chemical composition of Si/Au NCs considerably affects the efficiency of their ultrafast laser hyperthermia ability achieving ~10–20 °C heating for 10 min. The obtained findings will help with the preparation of NCs with both of the required chemical composition and plasmonic properties used for surface-enhanced sensing and laser-stimulated hyperthermia.

## Figures and Tables

**Table 1:** Binding energies (BE) of Au 4f and Si 2p core level peak components.

	BE Au 4f^7/2^, eV				BE Si 2p^3/2^, eV			
Time, min	Au_0_	Au-Si	Au^1+^	Au^3+^	Si_0_	Si^1+^	Si^3+^	Si^4+^/SiO_2_
0.0	---	---	---	---	99.1	100.7	102.7	103.7
0.5	---	84.9	85.5	86.4	99.2	100.8	102.8	103.8
1.0	---	84.6	85.2	86.1	99.0	100.7	102.7	103.7
1.5	84.0	84.5	85.2	86.0	99.2	100.7	102.7	103.7
3.0	83.9	84.5	85.1	85.9	99.1	100.7	102.7	103.7
5.0	84.0	84.5	85.2	86.0	99.4	100.8	102.8	103.8

**Table 2:** Parameters of Si/Au NCs aqueous colloidal solutions depending on the laser ablation time.

Ablation Time (min)	Au Mass (µg)	Au Content (%)	Plasmonic Intensity (a.u.)	Bandgap (eV)	Hydrodynamic Size (nm)	ξ-Potential (mV)	Concentration (10^11^ NPs/mL)	Conductivity (µS/cm)	Maximum Heating at 10 min (°C)
0.5	18	15	0.64	1.44	92.4	–42	4.96	2.04	18.1
1	42	29	0.84	1.39	85.5	–38	6.28	1.84	19.8
1.5	57	36	1.02	1.35	75.6	–35	7.37	1.67	17.5
2	66	40	1.35	1.32	73.9	–33	8.22	1.53	16.7
2.5	85	46	1.39	1.30	66.8	–32	8.66	1.67	13.0
3	115	53	1.64	1.28	58.2	–31	8.86	1.77	15.6
5	150	60	2.33	1.25	55.3	–30	9.63	1.92	12.5
7	208	68	3.02	1.24	52.1	–30	10.2	2.05	11.5
10	366	79	4.02	1.24	51.9	–30	10.3	2.38	11.0

## Data Availability

Data are contained within the article.
